# Comparing the Antinociceptive Effects of Methamphetamine, Buprenorphine, or Both After Chronic Treatment and Withdrawal in Male Rats

**DOI:** 10.32598/bcn.10.4.290.5

**Published:** 2019-07-01

**Authors:** Farshid Etaee, Arezoo Rezvani-Kamran, Mohammad Taheri, Ghazaleh Omidi, Parisa Hasanein, Alireza Komaki

**Affiliations:** 1. Neurophysiology Research Center, Hamadan University of Medical Sciences, Hamadan, Iran.; 2. Urogenital Stem Cell Research Center, Shahid Beheshti University of Medical Sciences, Tehran, Iran; 3. Department of Biology, School of Sciences, University of Zabol, Zabol, Iran.

**Keywords:** Methamphetamine, Buprenorphine, Pain, Hot plate, Tail flick, Interactions

## Abstract

**Introduction:**

Methamphetamine (Meth) and Buprenorphine (BUP) modulate pain perception. However, the antinociceptive effects of their interactions, which affect through different systems, are unclear in rats. This study aimed to compare the analgesic effects of Meth, BUP, and their coadministration, as well as the effect of withdrawal from these substances on nociception in male rats.

**Methods:**

In this experiment, 40 male Wistar rats (weight: 250–300 g) were categorized into four groups: control, Meth, BUP, or BUP+Meth. After seven days of treatments, the antinociceptive effects were assessed using the hot plate and the tail flick tests. The differences among the groups were analyzed with ANOVA and Tukey’s post hoc tests. P values less than 0.05 were considered significant.

**Results:**

Meth and BUP increased the reaction times during the hot plate and tail flick tests. The combination of Meth and BUP increased reaction time more than Meth or BUP alone.

**Conclusion:**

The significantly high reaction times in rats treated with Meth and BUP indicate that these substances have antinociceptive effects. In addition, Meth enhanced the antinociceptive effects of BUP. These synergistic effects might occur through the dopaminergic, serotonergic, and or adrenergic systems.

## Highlights

- There should be no debate about the need for new analgesic medications.
- Some studies reported that psychostimulant drugs could increase opioid-induced analgesia.
- We tested the analgesic effects of methamphetamine (Meth), Buprenorphine (BUP), and their coadministration in rats.
- The combination of Meth and BUP increased reaction time more than these two alone.
- Meth increases the analgesic effects of BUP.

## Plain Language Summary

There is no doubt about providing access to pain medications for those with a serious illness such as cancer, especially in a palliative care stage. Millions of people still lack access to drugs such as morphine, and millions more have access to ineffective drugs and continue to suffer from poorly-controlled symptoms. Additionally, because of some adverse effects of opioid medications, such as respiratory depression, especially in higher doses, we aimed to explore ways for increasing the analgesic effects of opiate medications without an increase in those adverse effects. Buprenorphine is approved as an opioid analgesic (painkiller) for various types of pain. In this study, we tested the analgesic effects of methamphetamine, buprenorphine, and their coadministration in rats to find new medications and compounds for effective controlling of pain in end-stage patients. We proved that methamphetamine decreases pain sensation in rats. Also, methamphetamine increases the analgesic effects of buprenorphine, and this combination can be used for more analgesic effects. Furthermore, our study results implicate that psycho-stimulant drugs, such as methamphetamine are good candidates for enhancing the analgesic effects of opioid medications.

## 1. Introduction

Pain is an unpleasant sensory and emotional experience that is associated with actual or potential tissue damage and is often accompanied by the desire to stop and avoid stimuli that cause it (Ripamonti, 2012). The perception of pain and its sensitivity to analgesics are highly variable (Bulka et al., 2004). Providing postoperative pain relief and analgesia is an essential step in pain management (Garimella & Cellini, 2013), and several different analgesics have been used for this purpose (Flecknell, Roughan, & Stewart, 1999).

Buprenorphine (BUP) is approved as an analgesic for various types of pain (Johnson, Fudala, & Payne 2005). It is a clinically well-established opioid analgesic (Christoph et al., 2005) that is currently used to treat opiate addiction and chronic pain (Browne, van Nest, & Lucki, 2015). BUP is a highly lipophilic derivative of oripavine (Cowan, Lewis, & Macfarlane, 1977); it is a partial agonistic for the μ receptor, an antagonist for the δ- and κ-opioid receptors, and produces limited euphoric effects (Lelong-Boulouard et al., 2006; Mori et al., 2006). It has a rapid onset and long duration of action in rodents. Because it is a partial μ-opioid agonist, it might have a more extensive safety profile than full μ-agonists, especially about respiratory depression (Johnson et al., 2005). The oral administration of BUP is both convenient and effective (Leach, Forrester, & Flecknell, 2010). Because it is 7–10 times more potent than morphine, the oral form may be an alternative to injected form of BUP for postoperative pain management (Jessen, Christensen, & Bjerrum, 2007).

In recent years, an increasing number of studies have examined the common mechanisms of reward and the analgesic effects of addictive substances. Therefore, the brain reward circuitry has been proposed as another key target for the pharmacological treatment of pain (Yamamotová et al., 2011). Psychostimulant drugs can increase opioid-induced analgesia (Dalal & Melzack, 1998b). Accordingly, drugs of abuse are known to have analgesic effects (Yamamotová et al., 2011). In this sense, opioid and psychostimulant drugs have long been used to relieve chronic pain in the clinics (Altier & Stewart, 1999). Methamphetamine (Meth) is a psychostimulant drug of abuse that acts on the central nervous system (Melo et al., 2012). It has a relatively high lipid solubility, that helps it to cross the blood-brain barrier (Yamamotová et al., 2011).

Determining the drug-induced changes in the reaction times of animals exposed to heat is the most widely-used measure of analgesic activity. Among the thermal methods, the hot plate and tail flick tests are most commonly used to assess opioid analgesia (Gades, Danneman, Wixson, & Tolley, 2000). We tested the antinociceptive effects of Meth, BUP, and their coadministration in Wistar rats with the hot plate and tail flick tests, to investigate enhancements of the antinociceptive effects of BUP. The present study investigated the use of psychostimulant drugs, including Meth, as an alternative for treating pain, instead of opioids such as BUP. We aimed to explore ways for increasing the antinociception of opiate drugs. In light of this, we tested whether Meth could increase the antinociceptive effect of BUP, how nociception was affected during withdrawal, and whether rats perceive pain differently in this state in comparison with the control animals.

## 2. Methods

### 2.1. Study animals

Adult male Wistar rats weighing 250–300 g were studied in this investigation. The animals were randomly arranged within four groups (Ten rats per each group); moreover, they were maintained on a 12:12 h light/dark program (lights on at 07:00 AM) within a temperature-controlled (22±2° C) place (Shiri et al., 2016).

The rats were fed ad libitum with standard chow-diet and water. Three days before the tests, the animals were housed in groups of four. All procedures of investigation and animal care were done according to the Veterinary Ethics Committee of the Hamadan University of Medical Sciences and the National Institutes of Health Guide for Care and Use of Laboratory Animals (NIH Publication No. 85-23, revised 1985).

### 2.2. Study drugs

Methamphetamine hydrochloride was obtained from the Presidency Drug Control Headquarters (Tehran, Iran). It was dissolved in 0.9% saline (Xu et al., 2015) and administered at a dose of 2 mg/kg (Miladi-Gorji, Fadaei, & Bigdeli, 2015; Etaee et al., 2017). BUP (Faran Shimi Pharmaceutical Co., Tehran, Iran) was dissolved in 0.9% saline (Wala & Holtman, 2011) and administered at a dose of 5 mg/kg (Thompson et al., 2006; Leach et al., 2010).

### 2.3. Study groups

In this experiment, 40 male rats were divided into the following four groups. The control group was administered saline by Intragastric (IG) gavage once a day for seven days. The Meth group was Intraperitoneally (IP) administered 2 mg/kg of Meth hydrochloride once a day (Chiang, Hung, & Ho, 2014) for seven days (Miladi-Gorji et al., 2015; Etaee et al., 2017). The BUP group was administered 5 mg/kg of BUP by IG gavage once a day for seven days (Wala & Holtman, 2011). Finally, the BUP+Meth group was administered BUP (IG; 5 mg/kg once a day for seven days [before Meth]) and Meth hydrochloride (IP; 2 mg/kg, once a day for seven days). On the day of behavioral testing, Meth and BUP were respectively administered 30 (Schutová, Hrubá, Pometlová, & Šlamberová, 2009) and 60 minutes (Wala & Holtman, 2011) before the tests. The withdrawal tests were conducted seven days after the first round of behavioral tests, which included the hot plate and the tail flick test. Both tests were conducted on the same rats.

### 2.4. Hot Plate Test

The hot plate device consisted of an electrically heated surface and an open Plexiglas tube (17 cm high×22 cm in diameter), which was used to confine the animals to the heated surface (Burj Sanat Co.). The rats were placed on the surface of the hot plate, which was maintained at 50±0.1°C, to induce noxious thermal stimuli (Taheri Azandaryani et al., 2015). Licking of the hind limb was noted as a nociceptive response (Shirafkan et al., 2013). The cut-off time was 30 s to avoid tissue damage (Bulka et al., 2004). The animals were tested after being treated with the drug/drugs once a day for seven days and then seven days after the abstinence period.

### 2.5. Tail Flick Test

To evaluate the antinociceptive effects, we used a tail flick apparatus (Burj Sanat Co.). The tip, base, and middle part of the tails of the rats were placed on a radiant heat source, which was set at 5 degree, and the reaction time of the animals was recorded. The mean value of three measurements was calculated and used in the analysis. The lamp intensity was 30% (Shirafkan, Sarihi, & Komaki, 2013). The tail flick latency was defined as the time (in seconds) for the rat to withdraw its tail from the radiant heat source. The cut-off time was 12 s to prevent tissue damage. The maximum possible antinociceptive effect would be induced when the animals did not show a tail flick reaction within the cut-off time (Christoph et al., 2005). The animals were tested after the drugs were administered once a day for seven days and then seven days after the abstinence period. The tail flick test was conducted after the hot plate test because the animals needed to be gently immobilized in a small Plexiglas restrainer during the measurements (Yamamotová et al., 2011).

### 2.6. Statistical analysis

The mean values of three measurements during the tail flick (time for the tip, base, and middle of the tail) and hot plate tests (time until hind limb licking) were calculated with computerized analyses. The differences between the groups were determined by 1-way Analysis of Variance (ANOVA), which was accompanied by the Tukey’s post hoc test. The differences with P values lower than 0.05 were considered significant. The data were expressed as the Mean±SD. We used the Student’s t-test to compare the results of the behavioral tests before and after seven days of abstinence.

## 3. Results

### 3.1. Hot Plate Test

**Figure 1.**
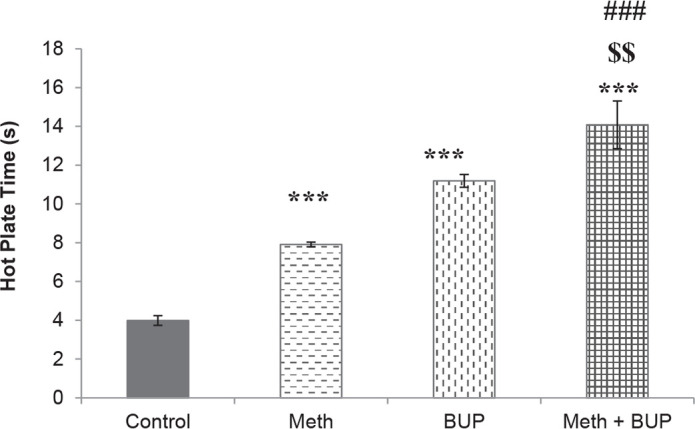
Effects of Meth (2 mg/kg, IP) and BUP (5 mg/kg, IG) administration on the reaction time during the hot plate test. ^***^P<0.001, for all groups in comparison to the control group; ^$$^P<0.01, for comparison of the BUP+Meth to the BUP group; ^###^P<0.001, for comparison of the BUP+Meth to the Meth group.

Meth administration significantly increased the reaction time during the hot plate test (the Meth group, 7.91±0.12 s; the control group, 3.99±0.25 s; P <0.001). The rats in the BUP group reacted slower (11.19±0.33 s) than those in the Meth (P <0.001) or control (P <0.001) groups. The coadministration of BUP and Meth resulted in a significantly longer (14.08±1.23 s) reaction time than the one induced by the single administration of BUP (P<0.01), Meth (P<0.001), or saline (P<0.001) (Figure 1).

### 3.2. Tail Flick Test

**Figure 2.**
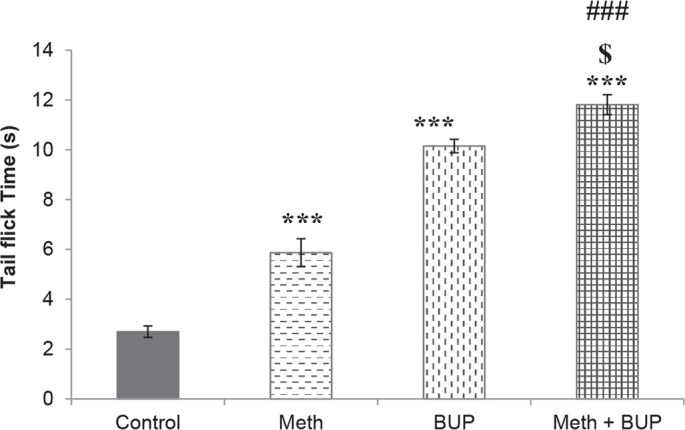
Effects of Meth and BUP on the latency time during the tail flick test ^***^P<0.001, for all groups in comparison to the control group; ^$^P<0.05, for comparison of the BUP+Meth to the BUP group; ^###^P<0.001, for comparison of the BUP+Meth to the Meth group.

Meth administration significantly increased the reaction time during the tail flick test (the Meth group, 5.87±0.56 s; the control group, 2.70±0.23 s; P<0.001). Rats in the BUP group showed a slower response (10.15±0.27 s) than those in the Meth (P<0.001) or control (P<0.001) groups. The coadministration of BUP and Meth resulted in a significantly longer reaction time of tail flick test (11.95±0.43s) than that obtained after a single administration of BUP (P<0.05), Meth (P<0.001), or saline (P<0.001) (Figure 2).

### 3.3. Hot Plate Test after seven days of drug abstinence

**Figure 3.**
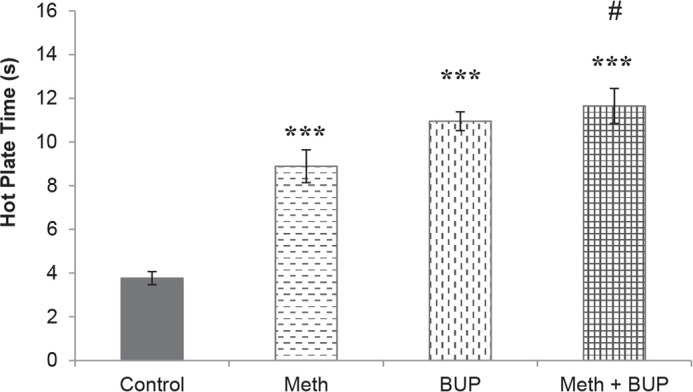
Effects of abstinence from Meth and BUP for seven days on the latency time during the Hot Plate Test ^***^P<0.001, for all groups in comparison to the control group; ^#^ P<0.05, for comparison of the BUP+Meth to the Meth group.

The abstinence from Meth, BUP (11.19±0.33 s), or both BUP and Meth (14.08±1.23 s) resulted in significantly higher hot plate latencies than the respective saline values (Meth, 7.91±0.12 s; BUP, 11.19±0.33 s; BUP+Meth, 14.08±1.23 s; saline, 3.99±0.25 s; P<0.001). Additionally, the combined withdrawal from BUP and Meth resulted in longer reaction times than that obtained after abstinence from Meth alone. No significant changes were seen among the other groups (P>0.05) (Figure 3).

### 3.4. Tail Flick Test after seven days of drug abstinence

**Figure 4.**
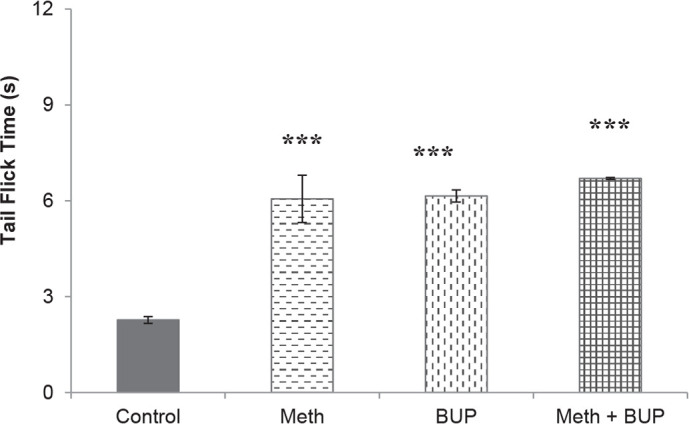
Effects of abstinence from Meth and BUP for seven days on the latency time during the tail flick test ^***^P<0.001, for all groups in comparison to the control group.

The abstinence from Meth, BUP and their combination significantly increased the tail flick test times in comparison to the respective saline values (Meth, 6.06±0.74 s; BUP, 6.15±0.19 s; BUP+Meth, 6.70±0.03 s; saline, 2.27±0.107 s; P<0.001). There were no significant changes in the reaction times of the other groups (P>0.05) (Figure 4).

### 3.5. Comparison of the Hot Plate Test reaction times between Meth and BUP treatment and after their withdrawal

**Figure 5.**
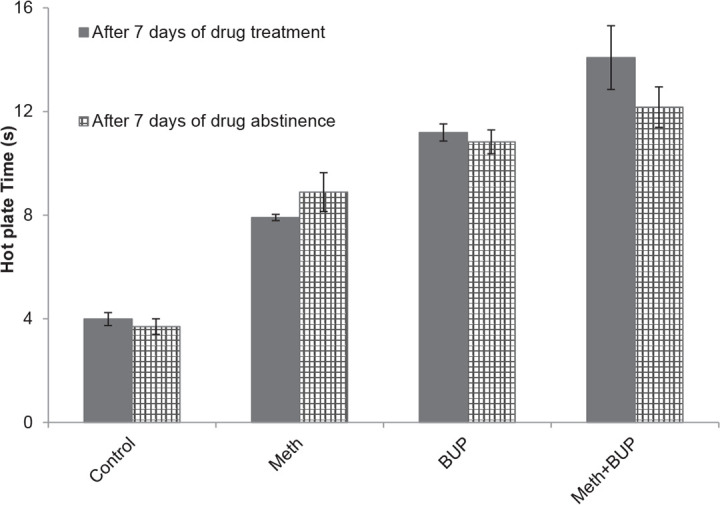
Comparison of the latency time during the hot plate test between the periods of Meth and BUP treatment and abstinence. P>0.05 for all groups

As revealed by t-test analysis, there were no significant differences between treatment and withdrawal from any of the drugs tested in the reaction times of the different groups (P>0.05) (Figure 5).

### 3.6. Comparing the reaction times of the Tail Flick Test between Meth and BUP treatment and after their withdrawal

**Figure 6.**
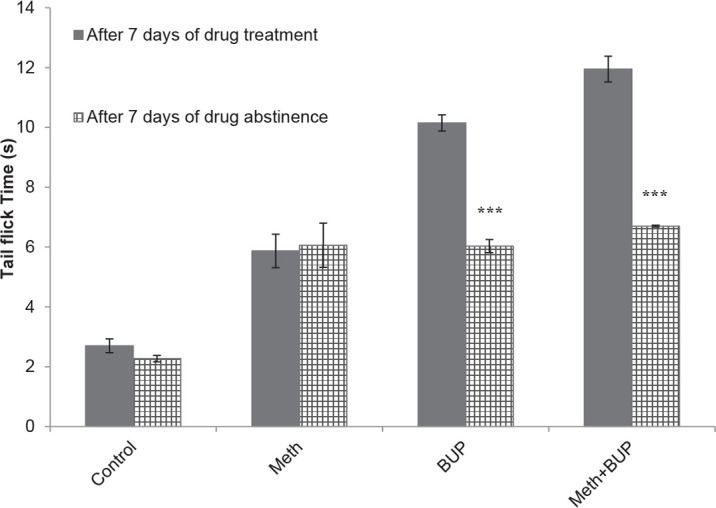
Comparison of the latency time during the tail-flick test between the periods of Meth and BUP treatment and abstinence. ^***^P<0.001, for the BUP and BUP+Meth groups.

The t-test analysis showed that the BUP and BUP+Meth groups exhibited significant decreases between treatment and after abstinence in the tail flick test reaction times (P<0.001 for both). No significant differences were observed between the Meth and control groups (P>0.05 for both) (Figure 6).

## 4. Discussion

The results of the two behavioral tests revealed that chronic injections of Meth in healthy rats significantly prolong their reaction time to the delivered stimulus compared to saline administration. Therefore, the dose of Meth used in this study exhibited antinociceptive effects. Besides, similar results were obtained in the case of BUP administration, indicating the analgesic effects of this drug, too. The coadministration of BUP and Meth resulted in an even more pronounced increase in the reaction times. Therefore, Meth enhanced the antinociceptive effects of BUP.

Our analyses showed that the latency times of tail flick test seven days after drug abstinence were significantly lower than those after seven days of drug treatment in the BUP and BUP+Meth groups. However, we did not detect any significant differences in the results of the hot plate test. The withdrawal from Meth, BUP, or BUP and Meth combined significantly increased the latency times in both behavioral tests. Therefore these drugs, during treatment or the state of abstinence, have analgesic effects.

In this study, BUP exhibited antinociceptive effects, that are in agreement with previous study results (Johnson, Fudala, & Payne, 2005). Also, our results revealed that chronic Meth injections induce antinociceptive effects in rats. Consistent with these findings, psychostimulant drugs have been reported to produce analgesic effects (Dalal & Melzack, 1998b) and potentiate opioid analgesia (Dalal and Melzack, 1998a). The analgesic and reinforcing effects of drugs of abuse are mediated by similar receptors, similar sites of action, and overlapping neural substrates. Recent studies have suggested that activation of the mesolimbic dopamine neurons that originate from the Ventral Tegmental Area (VTA) and extend to the Nucleus Accumbens (NAc) plays an essential role in mediating the suppression of tonic pain (Altier & Stewart, 1999). These similarities suggest that the reinforcing effects of these drugs may also produce analgesia by transforming the aversive affective states evoked by pain into more positive states (Franklin, 1998).

Central dopamine systems have been implicated in reward-related behavior (Bubenikova-Valesova et al., 2009). Partial agonists of μ-opioid receptors, such as BUP, increase the extracellular concentrations of dopamine in the NAc (Nantwi, Hicks, Bradley, & Schoener, 1998) and striatum when they are administered systemically or into the VTA or Substantia Nigra (SN) (Johnson & North, 1992; Chefer, Denoroy, Zapata, & Shippenberg, 2009). Accordingly, the dopaminergic neurons in the VTA that project to various forebrain sites, including the NAc, are involved in this process. The dopamine-containing neurons of the VTA play a critical role in the reinforcing effects of drugs of abuse, including opiates, and their turn-over in the NAc, suggesting that these effects are mediated by an increased output of dopamine (Nantwi et al., 1998).

Most of the afferents to the SN dopaminergic neurons are GABAergic, while dopaminergic neurons expressing GABA receptors and μ-opioid receptor mRNA are found both in the SN and VTA in rats (Mori et al., 2016). The opioid-induced release of dopamine in the NAc and striatum is probably caused by the inhibition of GABA interneurons, which subsequently stimulate the dopaminergic neurons (Chefer et al., 2009). Accordingly, the systemic administration of opiates has been shown to increase the firing of VTA dopamine neurons, as demonstrated by in vivo recordings (Johnson & North, 1992).

Substantial evidence indicates that psychostimulant drugs directly increase the levels of extracellular dopamine. In line with this finding, Meth has been reported to increase the release of dopamine (Yamamotová & Slamberova, 2012) and its extracellular concentration partly by reversing the dopamine transporter and depleting cytoplasmic as well as vesicular dopamine stores (Wallace, Gudelsky, & Vorhees, 1999). Consistent with these reports, the onset of Meth-induced analgesia, occurring 30 minutes after administration of the drug, correlates with the peak of the extracellular dopamine concentrations in the striatum. To understand the analgesic effects of psychostimulants, it is important to take into account that VTA neurons receive nociceptive information and are involved in pain modulation (Yamamotová et al., 2012). Psychostimulants and opioids both increase the extracellular concentrations of dopamine in the NAc (Mori et al., 2016).

Moreover, besides dopamine, Meth increases the levels of 5-hydroxytryptamine (serotonin) and norepinephrine in several brain regions in adult rats (Bubenikova-Valesova et al., 2009). Serotonin and norepinephrine are considered essential modulators of pain transmission, especially in the descending antinociceptive system (Jacobs et al., 2002). A large body of evidence implicates the serotonin pathway, especially the serotonergic neurons that are localized in the Nucleus Raphe Magnus (NRM) and that directly project to the dorsal horn of the spinal cord, in analgesia (Jacobs et al., 2002). Both opiate and stimulus-induced analgesia appear to depend on these descending connections to the spinal cord. NRM has been suggested to regulate the relief and the transmission of spinal pain induced by opiates or by stimulation of the periaqueductal gray (Basbaum et al., 1976).

The reinforcement of noradrenergic neurotransmission might, therefore, add to the efficacy of opioids, while, at the same time, norepinephrine uptake inhibitors have been shown to enhance the antinociceptive actions of systemically or centrally administered opioids in rats (Driessen, Reimann, & Giertz, 1993). The μ receptors, located at discrete and anatomically distant brain sites, mediate opioid peptide-induced catecholamine secretion through activation of the central sympathetic outflow to the adrenal medulla and sympathetic nerve terminals (Appel, Kiritsy-Roy, & Van Loon, 1986).

Increased extracellular norepinephrine increases pain thresholds by acting on α2-adrenergic receptors (Bohn, Xu, Gainetdinov, & Caron, 2000). The descending noradrenergic system and nociceptive system are closely related to the spinal cord of rats (Kuraishi, Hirota, Satoh, & Takagi, 1985). In addition, most psychostimulants increase norepinephrine neurotransmission (Drouin et al., 2002). Accordingly, the involvement of norepinephrine has been suggested in the arousal-promoting actions of psychostimulants (Berridge, 2008).

The results of the present study strongly support the hypothesis that psychostimulants, such as Meth, have analgesic effects and can increase the antinociception effects of opiate drugs. Based on previous investigations, we postulate that the dopaminergic, serotonergic, and noradrenergic systems perform important functions in the enhancement of the antinociceptive effects of BUP by Meth. Although Meth and BUP both increase the extracellular concentrations of dopamine in the NAc, serotonin in the NRM, and norepinephrine in the brainstem, their exact mechanisms of action should be further investigated to understand their different analgesic effects better.

Our study results implicate that psychostimulant drugs, such as Meth are good candidates for enhancing antinociceptive effects. This finding is crucial for reducing opiate drug doses and preventing their adverse effects while at the same time, enhancing their analgesic effects. Future studies are required to examine the effects of different doses, various routes of administration, and different treatment duration of these drugs.
